# Eletromyography of abdominal muscles in different physical exercises: An update protocol for systematic review and meta-analysis

**DOI:** 10.1097/MD.0000000000010395

**Published:** 2018-04-27

**Authors:** Thiago Montes Fidale, Felipe Farnesi Ribeiro Borges, Leonardo Roever, Gilmar da Cunha Souza, Alexandre Gonçalves, Eduardo Paul Chacur, Cristhyano Pimenta, Eduardo Gasparetto Haddad, Guilherme Gularte de Agostini, Fábio Clemente Gregório, Fabrício Cardoso Ribeiro Guimarães, Franciel José Arantes, Lázaro Antônio dos Santos, Adriano Alves Pereira, Hanna Karen Moreira Antunes, Guilherme Morais Puga, Frederico Balbino Lizardo

**Affiliations:** a Electromyography Laboratory of Institute of Biomedical Sciences; b Laboratory of Experimental Medicine, Federal University of Uberlândia, Uberlândia; c Department of Biological Sciences, Federal University of Goiás, Catalão; d UNIPAC College of Uberlândia, Uberlândia; e College of Physical Education, Federal University of Uberlândia, Uberlândia; f Atenas College Morphofunctional Department, Paracatu; g Departament of Bioscience of the Federal University of Sao Paulo, São Paulo, Brazil.

**Keywords:** abdominal exercises, electromyography, EMG, exercises, MIVC, rectus abdominis, RMS, strength exercises

## Abstract

**Background:**

The abdominal muscles are extremely important because they are directly involved in the functions of support, containment of viscera, and help in the process of expiration, defecation, urination, vomiting, and also at the time of childbirth. Many exercises and equipment are used to strengthen the abdominal muscles, and the workouts are proposed for a variety of purposes, such as preventing and rehabilitating low back pain, improving sports performance, achieving aesthetic standards, among others. Exercises that potentiate the electromyographic activity promote a greater recruitment of muscle fibers and are more effective to improve or maintain of the force. The electromyographic activity analysis allows us to reflect on the quality of the exercises proposed, consequently, to choose and order the exercises properly in a training session.

**Methods:**

Our systematic review protocol will developed following the reporting items for the systematic review. To identify relevant studies, we sought articles on the following bases: MEDLINE, PubMed, Europubmed, SciELO, Physiotherapy Evidences Data Base (PEDro), Cochrane, and Google Scholar. The methodological quality of the studies included in the review will evaluated using a checklist and quality assessment. For intervention studies, risk of bias will estimated using the Cochrane Collaboration tool.

**Results:**

The results of this study will show the electromyographic activation of the abdomen in the different types of exercises.

**Conclusion:**

Ethics approval was not required for this study because it was based on published studies. The results and findings of this study will be submitted and published in a scientific peer-reviewed journal.

**Systematic review registration:**

PROSPERO CRD42018086172.

## 1 Background

The abdominal muscles are extremely important because they are directly involved in the functions of support, containment of viscera, and help in the process of expiration, defecation, urination, vomiting, and also at the time of childbirth.^[1]^

The importance of the rectus abdominis muscle in the normal posture of the pelvis is emphasized. Besides that, it is an indirect responsible for the lumbar curvature and of great importance in the posture of the body.^[2]^ A weak abdomen causes several disorders associated with posture (such as ptosis or low back pain), respiratory disorders, and others.^[3]^

Exercises aimed to abdominal strengthening have been practiced, as they involve not only aesthetic goals, but also the prevention and/or rehabilitation of low back pain, improvements athletic performance, increase resistance, and strength of the torso for daily activities, and others.^[4,5]^

The higher the electrical activity of the muscle, larger amount of fibers recruited, and improves strength.^[6,7]^ Exercises that potentiate the electromyographic activity impose greater challenges on the neuromuscular system, therefore they are more effective for the improve the force capacity.^[8]^

The hypothesis is that there is a difference in muscle activity observed through the electromyographic values for the rectus abdominis muscle when comparing different exercises proposed to improve abdominal force, performed with and without the use of apparatus.

## 2 Objectives

This systematic review aims to analyze the activation of the abdominal muscles (rectus abdominis) according to results obtained through electromyographic analysis during the execution of different physical exercises.

## 3 Methods/design

Our systematic review protocol was developed following the reporting items for the systematic review. The review protocol was recorded in the International Prospective Review Record (PROSPERO) (registration number: CRD42018086172). This study will not involve any private patient data; ethics approval was waived (see online supplementary file 1 for PRISMA-P checklist).

### 3.1 Study design

**Figure 1:**
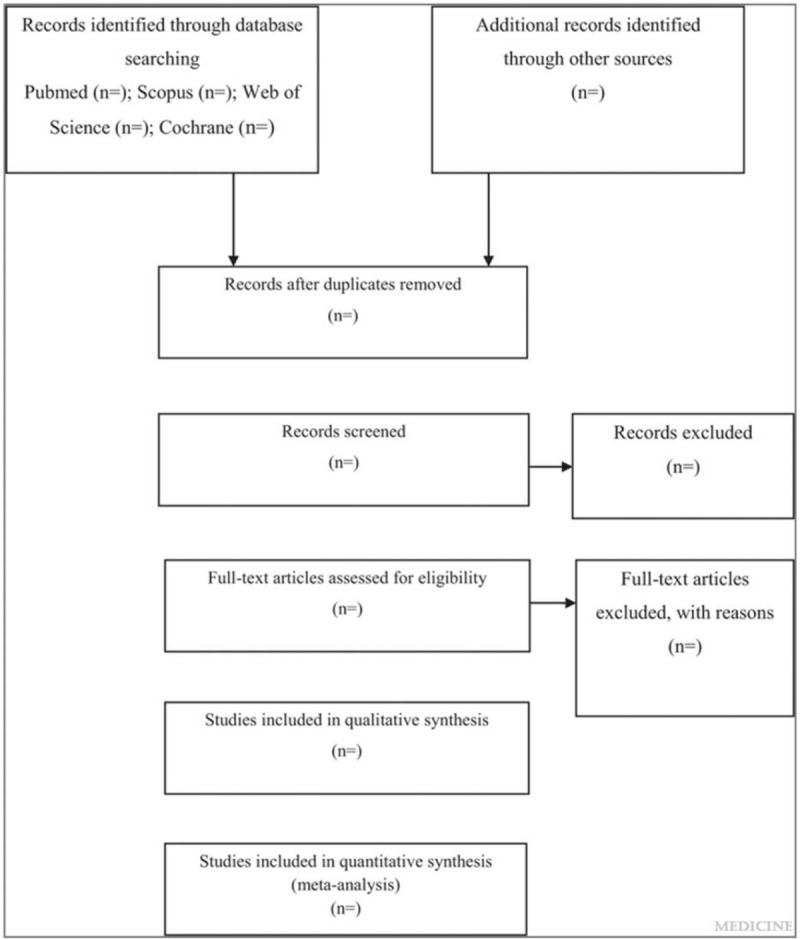
Flow diagram of study selection process.

This is a systematic review and meta-analysis protocol of prospective cohort studies, following the PRISMA-P (Preferred Reporting Items for Systematic Reviews and Meta-Analysis protocols) guidelines.^[9,10]^ The systematic review and meta-analysis will be reported according to the PRISMA (Preferred Reporting Items for Systematic Reviews and Meta-Analyses) guideline.^[11]^ The whole process of study selection is summarized in the PRISMA flow diagram (Fig. 1).

### 3.2 Inclusion/exclusion criteria

#### 3.2.1 Types of studies

Experimental, cross-sectional studies, case studies, observational studies, and randomized clinical control (RCT).

#### 3.2.2 Type of participants

The inclusion criteria were: studies conducted with healthy humans; population with age limit (18–60 years); trained and familiar subjects in the respective exercises studied. No restrictions of gender, ethnicity or socioeconomic status shall apply. The exclusion criteria are: studies performed with subjects presenting factors that may interfere with the reliability of the results sought, such as: pregnancy, fracture/displacement, osteoporosis, spine with malformation, skeletal deformities (e.g., Scheuermann kyphosis, scoliosis), leg length discrepancies, spondylitis/spondylolysis, rheumatic disorders (e.g., ankylosing spondylitis, rheumatoid arthritis), equine tail syndrome, abdominal surgery, infection, tumor, infection and systemic/cerebrovascular disease/neuromuscular diseases, heart disease, neurological dysfunctions, alcoholism, smoking, myopathies or neuromyopathies, low back pain, pain in the abdominal region, or any other type of clinical problem that could interfere in the execution of the exercises.

#### 3.2.3 Type of intervention

We will include interventions that investigated the electromyographic activation of the rectus abdominis muscle (RA), independently of its part, being URA (upper rectus abdominis) or LRA (lower rectus abdominis), also studies that present a percentage of maximal isometric contraction (% MVIC) and studies that performed the analysis of the electromyographic signal in the temporal domain.

#### 3.2.4 Types of measures of results

Types of studies that report the results of the percentage of maximal voluntary isometric contraction (% MVIC) and the analysis of the electromyographic signal in the temporal domain on the abdominal muscles in different types of physical exercise will be considered. Studies considered as outcome variable: do not evaluate the % MVIC, RMS or that do not study the electromyographic activation or that did not analyze the EMG signal in the time domain of the rectus abdominis muscle will be excluded. The data collection tools will include analysis of the results obtained through the use of the electromyograph in the exercises performed.

### 3.3 Search sources

#### 3.3.1 Electronic search

To identify relevant studies, the following electronic databases will be searched: MEDLINE, PubMed, Europubmed, SciELO, Physiotherapy Evidences Data Base (PEDro), Cochrane, Google Scholar. After analyzing the main studies and considering recommendations from specialists, the following keywords were identified for the study: MIVC, RMS, Rectus Abdominis, EMG, Electromyography, Strength Exercises, Abdominal Exercises, and Exercises (Table 1). Three authors will analyze the title and abstract of the articles found. Reference lists of relevant studies will be examined further for other potential studies to be included.

**Table 1:**
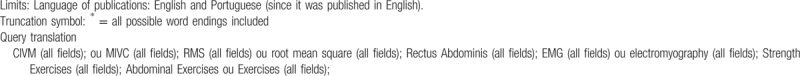
Sample search string for PubMed MEDLINE.

### 3.4 Data collection and analysis

#### 3.4.1 Data management

Duplicate articles will be removed, and the references evaluated for eligibility will be sorted alphabetically, according to the names of the first authors.

#### 3.4.2 Selection process

Two authors (TMF and FFRB) will independently analyze the titles and abstracts of studies identified by the research strategy. Potentially eligible studies will be re-evaluated by reading the full text. In case of disagreement, the opinion of a third author (FBL) will be requested. Following the guidelines (PRISMA-P), a flow diagram will illustrate the study selection process.

#### 3.4.3 Data collection process

The data will be extracted in ad hoc tables (Table 2). An author (TMF) will complete the extraction of data from selected studies (study design, year, country, number of participants, characteristics of volunteers, age, equipment used, exercise performed, treatment of RMS and% MIVC, obtained results and discussion). A second author (FFRB) will check the accuracy and consistency of all entries and make relevant clarifications when necessary. A third author (GCS) will arbitrate unresolved disagreements regarding data extraction.

**Table 2:**
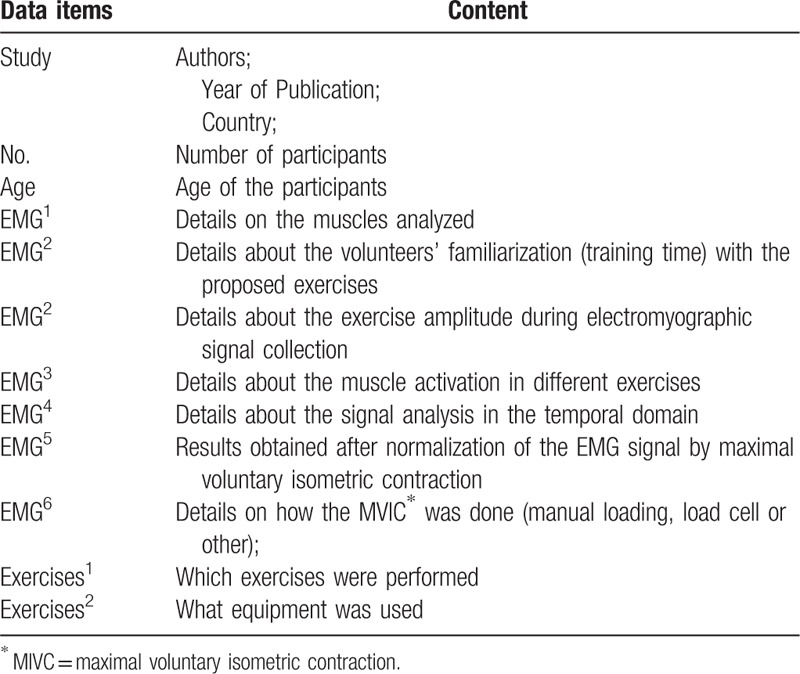
Data extraction variables.

#### 3.4.4 Assessment of bias quality and risk of included studies

Two independent reviewers will assess bias quality/risk using the Cochrane Handbook of Systematic Reviews of Interventions,^[12]^ thereby finding consensus through a discussion or a third party deciding whether no consensus has been reached. Will be checked: missing data, internal consistency of data, integrity of randomization (balance of volunteer characteristics at randomization, randomization pattern), standard of monitoring, and censorship. The summary tables will be verified with the test protocol and evaluation or publication report (Table 3). Any discrepancy or unusual patterns will be verified with the second reviewer of the study.

**Table 3:**
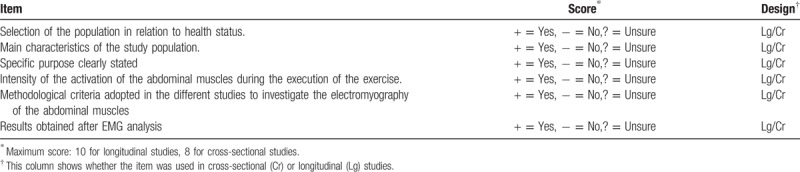
List of quality assessment of cross-sectional and longitudinal studies.

#### 3.4.5 Data synthesis

The ad hoc tables will demonstrate the summary of the data of the included studies and show their key characteristics and any important issues related to the purpose of this review. The *I*^2^ statistic will be used to evaluate statistical heterogeneity. Quantitative and narrative synthesis will be used to complete the review objective if a meta-analysis is not possible.

#### 3.4.6 Sensitive analysis

A sensitivity analysis will be performed excluding one-to-one analysis studies.

#### 3.4.7 Analysis of subgroups

Will not be performed.

## 4 Discussion

Previous studies have been published analyzing the muscular activity of exercises proposed for the strengthening of core muscles, however, no review article has been proposed within this theme.^[13–19]^ The present study will pioneer a centralized analysis of the activation of the abdominal muscles (rectus abdominis) during the execution of different exercises, thus providing a detailed summary of the available evidence. We will conduct and report our review using existing guidelines that will take into account the potential bias risks for each study.^[20]^

Electromyography is the gold standard for determining muscle action, so understanding the muscular activation generated by different physical exercises is of great importance for professionals related to health care, aesthetics and physical performance, since such information allows a reflection on the efficiency and safety of the proposed exercises, as well as important adjustments to an efficient prescription.^[21]^ This protocol is clear and well structured to maximize the extraction of relevant information, aiming to contribute with scientific information in summary form about the electromyographic activation of the abdominal muscles during the execution of the different physical exercises.

## Author contributions

TMF, FFRB, LR, GCS, AG, EPC, CP, EGH, GGA, FCG, FCRG, FJA, LAS, AAP, HKMA, GMP, and FBL conceived the study idea and devised the study methodology. LR, TMF, and FBL participated in the design and coordination of the study. TMF was primarily responsible for protocol writing and developed the search strategy. LR and TMF will screen identified literature, conduct data extraction and analyses the review findings. All authors read the drafts, provided comments and agreed on the final version of the manuscript.

**Conceptualization:** Thiago Montes Fidale, Felipe Farnesi Ribeiro Borges, Leonardo Roever, Gilmar da Cunha Souza, Alexandre Gonçalves, Eduardo Paul Chacur, Cristhyano Pimenta, Eduardo Gasparetto Haddad, Guilherme Gularte de Agostini, Fábio Clemente Gregório, Fabrício Cardoso Ribeiro Guimarães, Franciel José Arantes, Lázaro Antônio dos Santos, Adriano Alves Pereira, Hanna Karen Moreira Antunes, Guilherme Morais Puga, Frederico Balbino Lizardo.

**Methodology:** Thiago Montes Fidale.

**Supervision:** Frederico Balbino Lizardo.

**Writing – original draft:** Thiago Montes Fidale, Felipe Farnesi Ribeiro Borges, Leonardo Roever, Gilmar da Cunha Souza, Alexandre Gonçalves, Eduardo Paul Chacur, Cristhyano Pimenta, Eduardo Gasparetto Haddad, Guilherme Gularte de Agostini, Fábio Clemente Gregório, Fabrício Cardoso Ribeiro Guimarães, Franciel José Arantes, Lázaro Antônio dos Santos, Adriano Alves Pereira, Hanna Karen Moreira Antunes, Guilherme Morais Puga, Frederico Balbino Lizardo.

**Writing – review & editing:** Thiago Montes Fidale, Felipe Farnesi Ribeiro Borges, Leonardo Roever, Gilmar da Cunha Souza, Alexandre Gonçalves, Eduardo Paul Chacur, Cristhyano Pimenta, Eduardo Gasparetto Haddad, Guilherme Gularte de Agostini, Fábio Clemente Gregório, Fabrício Cardoso Ribeiro Guimarães, Franciel José Arantes, Lázaro Antônio dos Santos, Adriano Alves Pereira, Hanna Karen Moreira Antunes, Guilherme Morais Puga, Frederico Balbino Lizardo.
